# Beyond immersion: disentangling the technological and social drivers of visitor satisfaction in XR art exhibitions

**DOI:** 10.3389/fpsyg.2026.1793811

**Published:** 2026-04-01

**Authors:** Yuhan Chen, Mengxin Li

**Affiliations:** Department of Visual Communication Design, School of Art and Media, Sichuan Agricultural University, Ya'an, China

**Keywords:** art exhibitions, extended reality, revisit intention, simplified UTAUT2, technological affordances, viewing satisfaction

## Abstract

**Introduction:**

Although Extended Reality (XR) has fundamentally reshaped curatorial practices in art exhibitions, the psychological mechanisms underlying technology acceptance and revisit intention remain poorly understood. Current research has largely focused on hardware fidelity and passive sensory immersion, providing limited insight into the socio-psychological drivers of user experience and continuous visiting behavior.

**Methods:**

To address this, we developed and validated an integrated socio-technical framework that links XR technological capabilities (presence, immersion, and interactivity) with a simplified, context-adapted version of the Unified Theory of Acceptance and Use of Technology (UTAUT2), alongside multidimensional viewing satisfaction and revisit intention. We analyzed survey data from 387 participants with direct experience of XR art exhibitions using a two-step structural equation modeling (SEM) approach. First, confirmatory factor analysis was conducted for psychometric validation of the measurement model. Second, path analysis was employed to test the proposed hypotheses using a bias-corrected bootstrap approach to verify the mediation effects.

**Results:**

Our results showed that both XR technological affordances (*β* = 0.369, *p* < 0.001) and the simplified UTAUT2 framework (*β* = 0.391, *p* < 0.001) positively predicted viewing satisfaction, with socio-psychological factors showing marginally stronger predictive power. Interactivity and social influence emerged as core drivers, challenging the prevailing “isolationist paradigm” of head-mounted display-based XR. The simplified UTAUT2 exerted a significant partial mediating effect on the relationship between technological affordances and viewing satisfaction, while viewing satisfaction fully mediated the link between the UTAUT2 framework and revisit intention.

**Discussion:**

This study offers a more structured socio-technical explanation of XR user experience and provides practical guidance for immersive art exhibition practitioners seeking to enhance revisit intention. These findings extend existing theories by integrating socio-psychological factors with technological affordances, highlighting the importance of social influence in immersive exhibition contexts.

## 1 Introduction

Extended reality (XR) has been increasingly integrated into the artistic sphere, fundamentally shifting the visitor experience from the passive observation of static objects to the active habitation of volumetric, digital environments (Albrezzi, 2024; Dastane et al., 2025). Although this transition has redefined the museum visitor not merely as a viewer but as a co-creator within a dynamic system, it has introduced a complex sociospatial paradox. Specifically, since head-mounted displays (HMDs) offer a high level of sensory engagement by occluding the physical environment, they also risk creating an insular experience for the user, detached from the shared social context that characterizes the art museum experience. In an era of high-fidelity digital isolation, the question arises as to whether visitor satisfaction is driven more by the visual illusion of “being there” or by the social and agentic need to connect and interact.

Despite the rapid adoption of XR in curatorial practice, existing literature continues to exhibit a pronounced “technocentric” bias. Prevailing studies frequently prioritize hardware metrics, including but not limited to display resolution, tracking latency, and photorealism, as the principal determinants of user acceptance (e.g., Albrezzi, 2024; Lai et al., 2023; Lin et al., 2018). While these technical parameters elucidate the delivery of the image, they do not encompass the psychological processing of the experience. Current models frequently posit that heightened immersion invariably results in elevated satisfaction, overlooking that art appreciation is an inherently communicative and emotional endeavor rather than solely a sensory experience. Consequently, a theoretical gap exists concerning the translation of specific technological affordances (the functionality of the technology) into psychological acceptance (the user’s sentiments), particularly in non-utilitarian, hedonic settings such as art exhibitions.

To address this gap, the present study adopts an integrated socio-technical perspective on XR art exhibitions by linking technological affordances (i.e., presence, immersion, and interactivity) with a context-adapted Simplified Unified Theory of Acceptance and Use of Technology 2 (Simplified UTAUT2). This approach helps disentangle the specific drivers of visitor satisfaction and allows us to examine whether social validation remains influential even in physically isolating XR settings.

Guided by this objective, the present research sought to address three specific questions to clarify the mechanisms of XR art acceptance:

*RQ1:* In what ways do distinct technological affordances (presence, immersion, and interactivity) differentially affect the psychological acceptance of XR art?

*RQ2:* Considering the physically isolating nature of HMDs, to what extent does social influence affect visitor satisfaction in comparison to sensory factors?

*RQ3:* To what extent does the simplified UTAUT2 framework mediate the relationship between the technological features in question and the visitor's intention to revisit the site?

## 2 Literature review and hypothesis development

To explain the psychological underpinnings of XR art, user response should be defined in terms of two pivotal constructs: viewing satisfaction, conceptualized as an immediate cognitive-affective appraisal, and revisit intention, which denotes sustained behavioral engagement. Within the scope of media psychology, these concepts go beyond mere consumer preference; rather, they index the technology’s capacity to engender a “meaningful experience” by fulfilling the user’s immersive and social affordances.

### 2.1 Technological affordances of XR

Extended reality (XR) functions as an overarching taxonomy for interactive virtuality, wherein the variable “X” signifies both the range of technological modalities and their specific forms, such as Virtual Reality (VR), Augmented Reality (AR), and Mixed Reality (MR) (Dastane et al., 2025). Encompassing “Cross Reality,” XR is characterized by the synthesis of virtual and physical realms, producing environment-consistent phenomena via multimodal sensory stimulation—including visual, auditory, and haptic channels (Puig et al., 2020). Consequently, this ability to expand governs the user’s perceptual depth regarding three-dimensional spatiality (Billinghurst et al., 2015; Dastane et al., 2025; Rauschnabel, 2022).

Through the integration of Head-Mounted Displays (HMDs), high-precision tracking, and spatial audio, XR technology immerses users in digital environments, blocking out the physical world (Billinghurst et al., 2015). Conversely, AR and MR paradigms facilitate the seamless superposition of digital artifacts onto physical settings via spatial mapping and photometric consistency, thereby instilling a sense of physical presence within the hybrid space. Beyond visual fidelity, physics engines emulate physical laws, permitting direct manipulation of virtual entities through kinetic inputs. Concurrently, natural user interfaces (NUIs)—such as gestural and vocal commands—mitigate cognitive load and streamline interaction mechanics. This human–computer interaction (HCI) attributes reinforce user agency and refine the experiential quality through low-latency, deterministic feedback (Ho et al., 2025).

Within the XR scholarship, presence, immersion, and interactivity are established as distinct technological affordances governing the service experience (Billinghurst et al., 2015; Rauschnabel, 2022). Presence is an objective environmental attribute of XR, referring to the user’s perception that the virtual exhibition environment adheres to the cognitive logic and physical laws of the real world. Immersion is a subjective psychological state derived from presence. It is manifested when users focus their attention on the exhibition content, reducing their awareness of external distractions and technological mediation. Interactivity is an objective operational function of XR, denoting a bidirectional relationship between the user and the virtual environment or objects characterized by immediate and predictable feedback. Here, interactivity is an inherent technical function of XR art itself, rather than the hedonic motivation derived by users from the subjective psychological pleasure they experience (Ramírez-Correa et al., 2019). Collectively, these constructs determine the quality of the XR encounter (Jeon, 2023; Latoschik and Wienrich, 2022), a phenomenon bolstered by technological fidelity (Makransky and Lilleholt, 2018; Sundar et al., 2017). While empirical literature substantiates the capacity of XR to amplify satisfaction and engagement across sectors such as education, healthcare, and entertainment (e.g., Parsons, 2015; Radianti et al., 2020), research on the specific effects of these affordances on viewer experiences in art exhibitions is limited.

While these constructs are related in terms of lived XR experience, they refer to different aspects of that experience and are therefore treated as separate variables in this study. The properties and experiential consequences of the XR system itself can be described as follows: presence, immersion, and interactivity. The concept of presence pertains to the perceived reality and coherence of the virtual environment; immersion refers to the user’s attentional absorption in that environment; and interactivity refers to the extent to which the system allows responsive and meaningful action. In contrast, hedonic motivation pertains to the user’s positive emotional response to using XR, characterized by enjoyment, amusement, or satisfaction. In summary, the concept of technological affordances refers to the capabilities of a system, while hedonic motivation signifies the user’s valuation of the engagement mode. Therefore, we conceptualize them as related yet distinct constructs rather than amalgamating them into a single higher-order factor. Based on this, we propose the following hypothesis.

*H1:* The Technological Affordances of XR (Presence, Immersion, and Interactivity) positively influence viewing satisfaction in XR art exhibitions.

As a fundamental environmental attribute of XR technology, presence is rooted in the telepresence framework proposed by Slater and Wilbur (1997). It is realized through the integration of spatial presence (environmental authenticity) and embodied presence (a sense of self-location). This integration allows users to unconsciously identify with the virtual environment, thereby reducing their cognitive dissonance toward virtual exhibitions (Cummings and Bailenson, 2016). Furthermore, neuroscience research indicates that presence activates the brain’s default mode network (Slater and Sanchez-Vives, 2016), enabling XR to generate an esthetic phenomenology that surpasses that of traditional media. This enables users to perceive virtual art experiences as meaningful. Based on this theoretical grounding, we hypothesized H1-1: Presence positively influences viewing satisfaction in XR art exhibitions.

Immersion is a user-centered psychological state that emerges when users’ intrinsic psychological needs are satisfied and is derived from high-quality presence. Subsequently, it evolves into the typical flow state characterized by an accelerated perception of time and a detachment from external stimuli (Radu, 2014). In XR art exhibitions, this immersive state enhances the depth of user engagement by optimizing the allocation of cognitive resources. Even when users are aware of the technological mediation, they maintain high levels of attention toward the artistic content, deepening aesthetic appreciation and increasing viewing satisfaction. Therefore, this study proposed H1-2: Immersion positively influences viewing satisfaction in XR art exhibitions.

Interactivity is the core operational function that distinguishes XR art exhibitions from traditional physical exhibitions. It serves as the fundamental principle of human–computer interaction within XR systems (Regenbrecht and Schubert, 2021). XR art exhibitions utilize intuitive interaction methods that empower users to actively manipulate virtual artworks and perceive the exhibition environment through multiple senses. This enhances audiences’ engagement and fosters emotional resonance between users and virtual artworks (Ho et al., 2025; Szubielska and Imbir, 2022). Such an active agency reduces the psychological distance between users and artworks, personalizing and deepening the art appreciation experience and thereby improving viewing satisfaction. Therefore, H1-3 posits: Interactivity positively influences viewing satisfaction in XR art exhibitions.

### 2.2 Adapted technology acceptance (simplified UTAUT2)

The acceptance of new technologies is a critical factor influencing user satisfaction. The higher the level of technology acceptance, the stronger the satisfaction with and willingness to continuously use it (Dwivedi et al., 2019). In XR art exhibitions, the presence, immersion, and interactivity enabled by new technologies contrast sharply with the static viewing mode of traditional art shows, directly affecting visitors’ technology acceptance processes and satisfaction evaluations. To explicate visitor processing of the XR experience, this study employed the Unified Theory of Acceptance and Use of Technology 2 (UTAUT2), proposed by Venkatesh et al. (2012). UTAUT2 extends the original UTAUT framework by introducing consumer-specific constructs to account for voluntary technology adoption.

UTAUT2 has been extensively utilized in the domain of consumer technology research. However, numerous studies have demonstrated that not all of its original constructs possess equivalent salience across various settings. Research on hedonic systems suggests that users do not approach pleasure-oriented technologies in the same way as utilitarian ones, and that enjoyment-related factors may be more central than usefulness-based evaluations (Van Der Heijden, 2004). In addition, UTAUT2 has been applied in leisure-oriented settings, such as mobile online games (Ramírez-Correa et al., 2019), and review evidence shows that some constructs, especially price value, are often omitted when they are less relevant to the focal context (Tamilmani et al., 2021). A comparable resolution was arrived at by Schomakers et al. (2022), who opted against incorporating price value in their UTAUT2-based study due to the predominance of complimentary mHealth applications in their designated context.

In the context of this line of study, the present study employed a simplified UTAUT2 model for the specific context of XR art exhibitions. The primary focus of this study is not on the private ownership of XR devices or the routine personal use of technology, but rather on the exhibition-based cultural consumption of such devices. In this context, visitors typically do not evaluate XR in terms of device purchase value, and the equipment, technical support, and interaction environment are primarily provided by the hosting institution. Consequently, price value and facilitating conditions were deemed less significant in the present study. Conversely, the roles of performance expectancy and effort expectancy may be diminished in XR art exhibitions, where the fundamental purpose of participation lies not in task performance but in esthetic and experiential engagement. Consequently, the study retained social influence, hedonic motivation, and habit as the acceptance variables that are more directly related to voluntary participation in this setting.

Conversely, this does not imply that the omitted constructs are unimportant in all XR contexts. However, their relevance persists in the context of studies examining first-time users, domestic utilization of XR, or scenarios characterized by significant variations in cost, technical assistance, and access criteria among users. The simplified UTAUT2 model employed in this study is therefore best understood as a context-specific alternative for the present research, rather than as a general replacement for the full UTAUT2 model. Accordingly, we proposed the following hypothesis.

*H2:* The simplified UTAUT2 model positively influences viewing satisfaction in XR art exhibitions.

Social influence delineates the normative pressure exerted by reference groups on technology adoption. Within the realm of artistic expression, this construct extends beyond conventional peer pressure, representing a collaborative process of semiotic construction. Art appreciation is inherently a communicative act; its esthetic value emerges from collective validation rather than solitary observation (Cialdini and Goldstein, 2004; Venkatesh et al., 2012). This dynamic becomes critical within Extended Reality (XR), where the utilization of Head-Mounted Displays (HMDs) often induces physical isolation. Here, social influence counteracts atomization by fostering shared intentionality—the drive to align psychological states with those of others. Consequently, peer endorsement establishes a framework of “intersubjectivity,” attenuating cognitive uncertainty within virtual environments and priming users to interpret the experience as socially significant. Accordingly, H2-1 states: Social influence positively influences viewing satisfaction in XR art exhibitions.

Hedonic objectives centered on esthetic pleasure underpin art appreciation (Fingerhut and Prinz, 2018). Within digital exhibitions, technological sensory stimulation and interactivity serve as primary determinants of satisfaction, where innovation augments engagement through novelty. Specifically, XR technology elicits deep affective responses via multisensory immersion, thereby amplifying the hedonic dimension of the viewing experience. Therefore, we formulated H2-2: Hedonic motivation positively influences viewing satisfaction in XR art exhibitions.

Habit in this study refers to a routinized and internalized tendency to use a technology repeatedly over time. In information systems research, habit is commonly understood as a learned tendency that develops through repeated prior behavior and becomes partly automatic in later use (Limayem et al., 2007; Gikas and Grant, 2013). Within the UTAUT2 framework, habit is also recognized as a significant predictor of consumer technology utilization and continuance (Venkatesh et al., 2012). In this sense, the habit construct used here follows the broader UTAUT2 measurement tradition, in which repeated-use tendency may be expressed through item wording that extends beyond narrow automaticity alone. Within the domain of XR art exhibitions, habit is defined as a learned tendency to engage with XR as a familiar mode of viewing, rather than as a manifestation of clinical dependence or external compulsion. On this basis, we proposed H2-3: Habit positively influences viewing satisfaction in XR art exhibitions.

Correspondingly, user acceptance is contingent upon the specific technological affordances inherent to the system. Given that technology adoption and decision-making are underpinned by a synergy of cognitive evaluation and emotional experience (Makransky and Lilleholt, 2018; Slater and Sanchez-Vives, 2016; Venkatesh et al., 2012), we proposed the following hypothesis.

*H3:* The Technological Affordances of XR positively influence Simplified UTAUT2.

Presence mitigates technological anxiety by establishing a cognitive assimilation mechanism that facilitates the user’s perception of virtual elements (Skarbez et al., 2018). Specifically, heightened states of presence stimulate the brain’s reality-monitoring network, thereby triggering an unconscious acceptance of technological mediation (Sanchez-Vives and Slater, 2005). H3-1 therefore states: Presence positively influences Simplified UTAUT2.

Sustained immersion attenuates external cognitive load by optimizing the allocation of cognitive resources (Zhong et al., 2021). By diminishing the user’s awareness of the underlying technology, immersive states render interaction requirements more intuitive and less burdensome, subsequently increasing the propensity for technology acceptance (Huang et al., 2020). We proposed H3-2 as follows: Immersion positively influences Simplified UTAUT2.

Natural interaction modalities, such as gesture and gaze tracking, bridge the gap between user intent and system response, thereby fostering intuitive operations (Corrales-Paredes et al., 2023). Furthermore, the superior reliability of body motion mapping that distinguishes direct manipulation from abstract controller input substantially alleviates perceived technological complexity, rendering novel systems more approachable (Jacob et al., 2008; Shaer, 2009; Uz-Bilgin and Thompson, 2022). Thus, we proposed H3-3: Interactivity positively influences Simplified UTAUT2.

### 2.3 Viewing satisfaction and revisit intention

Satisfaction with the viewing experience is best understood here as a post-experience evaluation that shapes revisit intention. In the field of continuance literature, satisfaction has been identified as a pivotal factor in the relationship between actual use experience and continued intention (Bhattacherjee, 2001). Recent research on VR art exhibitions has similarly demonstrated that satisfaction plays a pivotal role in shaping users’ continuance intention after immersive exhibition experiences (Cheng et al., 2024). Concurrently, research in the domain of museum experience suggests that visitor experience is not one-dimensional, but is shaped by different aspects of the encounter, including interpretation, affective response, and interaction with the exhibition environment (Chen and Shang, 2021; Ceccarelli et al., 2024). In consideration of the aforementioned background, the present study conceptualizes viewing satisfaction in XR art exhibitions as a context-specific adaptation of post-experience satisfaction, rather than as an entirely novel construct. Specifically, functional satisfaction is defined as visitors’ evaluation of the quality and fluency of interacting with XR during the exhibition. Emotional satisfaction is defined as their affective and esthetic response, and physical satisfaction is defined as bodily comfort and the absence of XR-related discomfort. In this sense, the tripartite structure cannot be considered a simple relabeling of existing constructs. Instead, it adapts established satisfaction logic to the XR exhibition setting and extends it modestly by making physical comfort explicit, given the embodied demands and possible side effects of XR use (Chang et al., 2020).

Although functional, emotional, and physical satisfaction capture different aspects of post-experience evaluation, they are modeled here as first-order factors loading onto a second-order Viewing Satisfaction construct because they represent interrelated judgments formed from the same exhibition encounter. In XR art exhibitions, visitors do not usually separate technical fluency, affective resonance, and bodily comfort into wholly independent evaluations. Rather, these dimensions jointly shape an overall appraisal of whether the viewing experience was satisfactory. In this sense, the three dimensions are treated as distinct but reflective manifestations of a broader post-experience satisfaction construct. Accordingly, we conceptualized viewer satisfaction in XR as a multidimensional construct comprising functional, emotional, and physical dimensions, which synergistically influence the propensity to return. Thus, we posited:

*H4:* Viewing satisfaction has a positive effect on the revisit intention of XR art exhibition visitors.

Consequently, these three dimensions should be comprehended as post-experience evaluation outcomes, as opposed to substitutes for the utilitarian construct omitted in UTAUT2. Performance expectancy, effort expectancy, and facilitating conditions focus on expectations formed by users before or during use; whereas functional, emotional, and physical satisfaction focus on how audiences evaluate the experience after participation. This distinction is of particular relevance in the context of this study, as our primary interest lies not in the initial perception of ease of use among users of XR, but rather in their subsequent evaluation of the exhibition experience in terms of its overall quality, significance, and comfort (Bhattacherjee, 2001; Chang et al., 2020; Cheng et al., 2024).

Based on the aforementioned conceptual framework, functional satisfaction directly reflects the quality of the XR technology experience in art exhibitions. When XR technology operates smoothly, when the interactive functions align with the audience’s artistic viewing needs, and when the spatial mapping is precise, the audience’s cognitive load is reduced, thereby fostering sustained user engagement (Bhattacherjee, 2001). This positive technological experience will further encourage visitors to return. Accordingly, we proposed H4-1: Functional satisfaction positively correlates with the revisit intention of XR art exhibition visitors.

The core of emotional satisfaction lies in the emotional resonance and esthetic pleasure evoked by XR exhibition art. Unlike the satisfaction derived from technology or physiological experiences, emotional fulfilment stems from the profound emotional responses generated when visitors interact with virtual artworks (Fingerhut and Prinz, 2018). When XR technology enhances emotional immersion through multimodal stimuli, like 360° spatial audio and haptic feedback, audiences experience intense emotional states, such as awe, joy, and psychological restoration (Godley et al., 2025). This unique emotional imprint fosters lasting memories and sparks a desire to revisit these positive feelings. Therefore, we formulated **H4-2:** Emotional satisfaction has a positive effect on the revisit intention of XR art exhibition visitors.

Physical satisfaction reflects the physiological comfort experienced when using XR hardware in art exhibitions. Research indicates that physical discomfort, such as cyber sickness and fatigue, is a key barrier to sustained technology adoption (Rebenitsch and Owen, 2016). When visitors encounter minimal physical discomfort during an exhibition, negative emotions are mitigated, leading to improved overall evaluations of the experience and heightened willingness to revisit. Thus, H4-3 proposed: Physical satisfaction has a positive impact on the revisit intention of XR art exhibition visitors.

**Figure 1:**
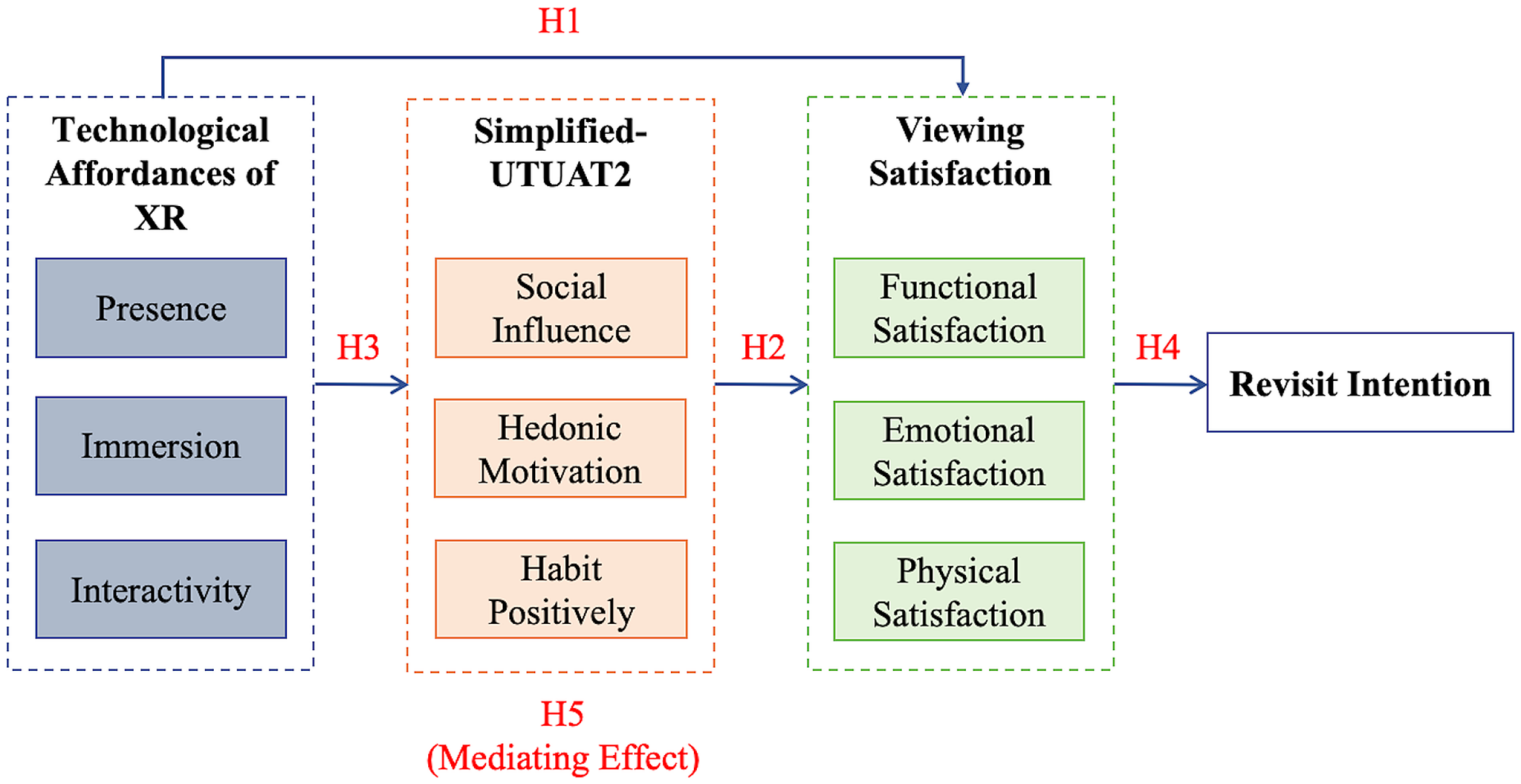
Conceptual model and hypotheses.

Synthesizing the deductive logic of H1 (Technological Affordances predicting satisfaction), H2 (Simplified UTAUT2 predicting satisfaction), and H3 (Technological Affordances predicting Simplified UTAUT2), we postulated that the Simplified UTAUT2 model functions as an intermediary mechanism. Specifically, it bridges the relationship between technological affordances and viewing satisfaction. The resultant hypothesis is illustrated in Figure 1:

*H5:* Simplified UTAUT2 mediates the relationship between Technological Affordances of XR and viewing satisfaction.

## 3 Materials and methods

### 3.1 Measurement development

To empirically validate the proposed conceptual framework and associated hypotheses, this study utilized a quantitative research design incorporating a rigorous two-wave sampling protocol. Recruitment occurred via mixed channels—online (Credamo) and offline—to ensure respondent validity. The initial wave functioned as a screening mechanism to isolate participants with authentic extended reality (XR) exhibition experience, thereby filtering out ineligible respondents before the administration of the core instrument in the second wave. The first-stage questionnaire focused only on respondents’ prior XR-related art exhibition experience, including whether they had participated in similar exhibitions and how frequently they had done so. This first-stage survey was used to build the participant pool for the second-stage survey. In the second-stage questionnaire, several experience-related questions were repeated, and responses across the two stages were compared to screen out cases with inconsistent or uncertain exhibition experience reports, thereby improving response quality. The resulting psychometric instrument consisted of 44 items structured into three distinct modules: exhibition metrics (history and frequency), core constructs (Technological Affordances, Simplified UTAUT2, Satisfaction, and Revisit Intention), and demographic variables. All constructs were measured on a 5-point Likert scale ranging from 1 (strongly disagree) to 5 (strongly agree).

**Figure 2:**
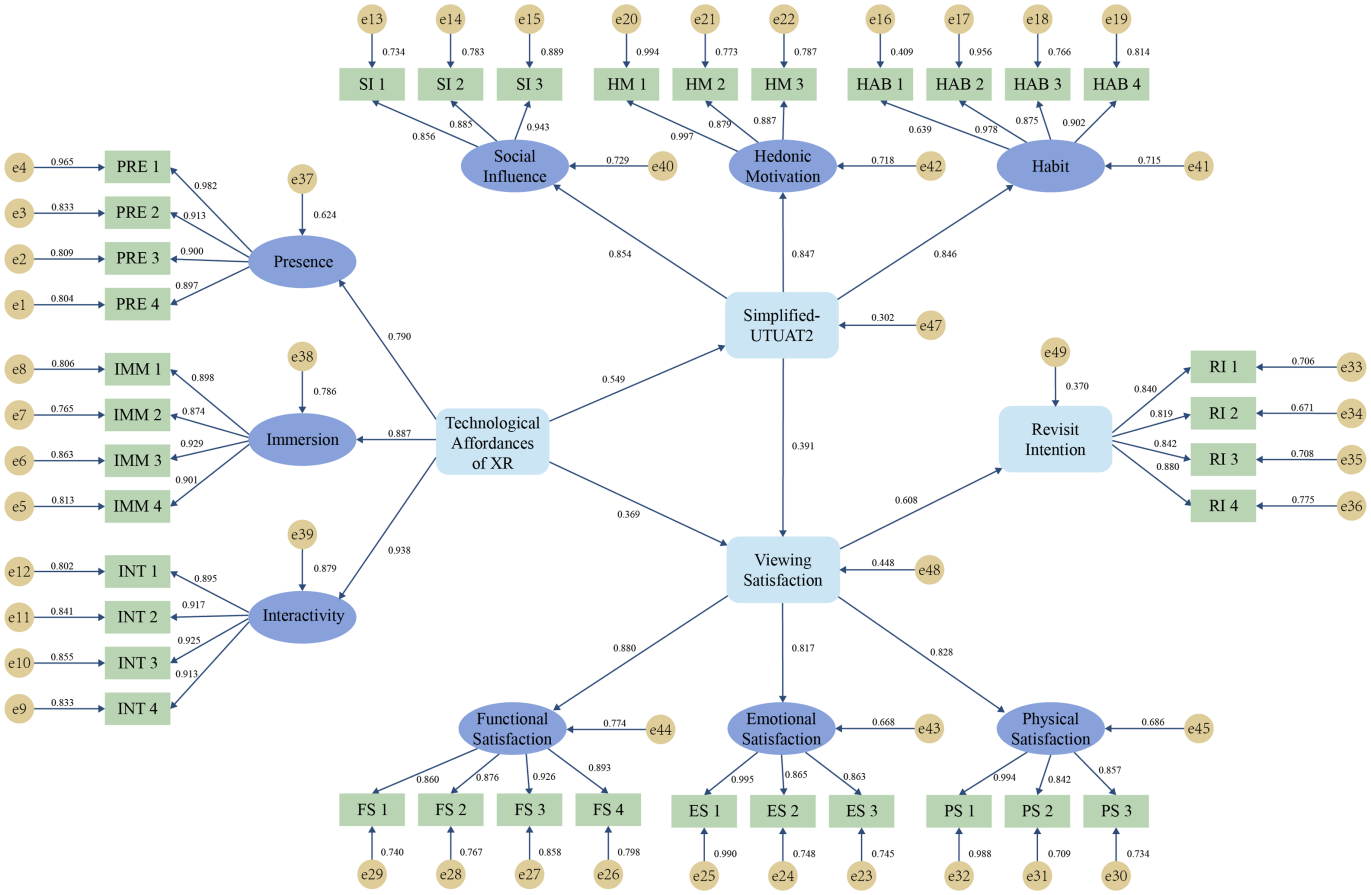
Path analysis of the research model.

The questionnaire used in this study was administered in Chinese. Most measurement items were adapted from previously published English-language scales (Table 1) and translated into Chinese by the authors. For some UTAUT2-related items, the Chinese wording also referred to formulations used in prior Chinese-language research to improve naturalness and readability in the present context. After the initial translation, the questionnaire was reviewed by other members of the research team, who checked the wording and conducted a back-translation to improve semantic consistency between the Chinese and English versions (Figure 2).

**Table 1:** Measurement items.

Constructs		Items	Contents	References
Technological affordances (TA)	Presence (PRE)	PRE 1	I was able to clearly perceive the three-dimensional spatial character of the virtual art exhibition, rather than just as a two-dimensional image on a flat screen.	Dahlkvist (2022), Gandy et al. (2010), and Slater and Sanchez-Vives (2016)
		PRE 2	The experience of realism that comes from viewing the exhibition makes me feel that the virtual characters/objects are real.	
		PRE 3	There was no confusion in the XR Art Exhibition about things that did not fit into the environment.	
		PRE 4	I can clearly perceive what is happening in the XR Art Exhibition.	
	Immersion (IMM)	IMM 1	I’m more focused on the art show itself than on any outside distractions.	Dahlkvist (2022), Georgiou and Kyza (2017), Lessiter et al. (2001), and Slater and Sanchez-Vives (2016)
		IMM 2	I was curious about how the exhibit was progressing.	
		IMM 3	I found myself easily forgetting that I was viewing the exhibit while using the XR technology.	
		IMM 4	The time passed quickly during the viewing event.	
	Interactivity (INT)	INT 1	I felt like I could touch the virtual objects in the exhibition.	Dahlkvist (2022), Gandy et al. (2010), Georgiou and Kyza (2017), and Regenbrecht and Schubert (2021)
		INT 2	I could perceive the interior environment of the art exhibition through my five senses (sight, hearing, smell, etc.).	
		INT 3	I often get excited because I feel like I’m part of the exhibition.	
		INT 4	I was very involved in the exhibition, and sometimes I wanted to interact directly with the virtual characters/objects.	
Simplified UTAUT2 (SU)	Social influence (SI)	SI 1	The people who matter to me think I should use XR technology for art exhibitions.	Chen and Liu (2023) and Ramírez-Correa (2019)
		SI 2	People who influence my behavior think I should use XR technology to view art exhibitions.	
		SI 3	People whose opinions I value prefer that I use XR technology to view art exhibitions.	
	Hedonic motivation (HM)	HM 1	It was fun to watch the art show using XR technology.	
		HM 2	Viewing an art exhibition using XR technology is relaxing.	
		HM 3	Art exhibitions using XR technology are very interesting.	
	Habit (HAB)	HAB 1	Using XR to view art exhibitions has become a habit for me.	
		HAB 2	I’m addicted to using XR technology to view art exhibits.	
		HAB 3	I must use XR technology to view art exhibitions.	
		HAB 4	Using XR technology to view art exhibitions has become natural to me.	
Viewing satisfaction (VS)	Functional satisfaction (FS)	FS 1	I found the XR technology used in the art exhibition easy to operate and smooth.	Billinghurst et al. (2015), Jennett et al. (2008), Jung et al. (2015), Leder and Nadal (2014), Nicodemus (2013)
		FS 2	I think the use of XR technology in the exhibition clearly conveys the core message of the artwork.	
		FS 3	I think the XR interactive feature in the exhibition can significantly enhance the fun during the visit.	
		FS 4	I found the interactive content provided by the XR technology in the exhibition to be highly compatible with my viewing needs.	
	Emotional satisfaction (ES)	ES 1	I had a great time using XR technology at the art exhibition.	Jung et al. (2015), Leder and Nadal (2014), and Slater and Sanchez-Vives (2016)
		ES 2	I would recommend XR art exhibitions to my friends.	
		ES 3	The XR technology inspired me to want to learn about and participate in more art exhibitions.	
	Physical satisfaction (PS)	PS 1	I did not feel any significant discomfort during my visit to the XR art exhibition.	Billinghurst et al. (2015), Guo et al. (2019), and Richard and Oliver (1980)
		PS 2	I was not kept in a fixed position for a long period of time during the XR art exhibition.	
		PS 3	I did not feel tired during the XR art exhibition.	
Revisit intention (RI)		RI 1	I’d love to go to an XR art exhibition in the future.	Bhattacherjee (2001), Cobb et al. (1999), and Pelowski et al. (2017)
		RI 2	I always make art shows that use XR technology my first choice.	
		RI 3	If I could, I would go to this kind of XR art show again.	
		RI 4	I would go to XR art shows with more companions.	

Before the formal survey was launched, the questionnaire was pretested in two stages. First, the authors completed the questionnaire themselves and discussed wording clarity and contextual appropriateness. Second, 10 participants from different age groups were invited to complete the questionnaire and provide feedback. Based on this process, minor wording adjustments were made to improve readability and comprehension. For transparency, the original Chinese items of the scale are provided in Supplementary Table S1.

Data collection spanned from March to June 2025. From an initial pool of 450 participants, 420 advanced to the core survey. Following a stringent data cleaning process that eliminated incomplete submissions and longitudinal inconsistencies regarding XR art exhibition history, a final dataset of 387 valid responses was retained. To verify sample sufficiency, a post-hoc power analysis using G*Power 3.1 confirmed that this sample size (N = 387) yields statistical power exceeding 99% (1-*β* > 0.99) for detecting a medium effect size (f^2^ = 0.15) at *α* = 0.05. Furthermore, the study protocol underwent ethical review before field implementation to ensure compliance.

### 3.2 Data collection and analysis

To verify the theoretical model and hypotheses, this study employed structural equation modeling (SEM) using AMOS 26.0 software. The analysis followed a two-step approach consistent with SEM best practices (Anderson and Gerbing, 1988). First, Confirmatory Factor Analysis (CFA) was conducted to assess the reliability and validity of the measurement model and ensure the psychometric quality of the latent constructs. Structural path analysis was then performed to test the direct relationships between constructs (testing all main and sub-hypotheses). To test the mediating effect (Hypothesis H5), the bootstrap method [5,000 resamples, 95% confidence interval (CI)] was employed to circumvent violations of normal distribution assumptions.

## 4 Results

### 4.1 Profile of participants

Table 2 presents the demographic characteristics of participants included in the second round of questionnaires. The sample consisted of 254 women (65.6%) and 133 men (34.3%). The participants were predominantly from two age groups: 18–30 years old (162 participants, 41.8%) and 31–40 years old (173 participants, 44.7%). The age group of 41–50 years old accounted for 8.3% (32 participants), and the group aged 51 and above accounted for 5.1% (20 participants). Regarding the annual number of visits to exhibitions (including traditional art exhibitions), 44.7% of the respondents (173 participants) visited 1–3 times a year, 36.2% (140 participants) visited 4–6 times, 13.7% (53 participants) visited 7–9 times, and 5.4% (21 participants) visited 10 times or more. Finally, in terms of the number of visits to XR art exhibitions, 57.1% of the respondents (221 participants) visited 1–3 times, 29.5% (114 participants) visited 4–6 times, 7.2% (28 participants) visited 7–9 times, and 6.2% (24 participants) visited 10 times or more.

**Table 2:** Demographics of the respondents.

Demographics		Respondents (%)	
Gender	Female	254	65.6
	Male	133	34.3
Age	18–30	162	41.8
	31–40	173	44.7
	41–50	32	8.3
	≥51	20	5.1
Exhibition frequency (Times/year)	1 ~ 3	173	44.7
	4 ~ 6	140	36.2
	7 ~ 9	53	13.7
	≤10	21	5.4
Number of visits to the XR art exhibition (Times)	1 ~ 3	221	57.1
	4 ~ 6	114	29.5
	7 ~ 9	28	7.2
	≤10	24	6.2

Given that the initial questionnaire was utilized for the purpose of screening respondents who had experience of XR-related art exhibitions, it can be posited that the present sample should be conceptualized as an audience of XR exhibitions with pertinent experience, as opposed to a general museum visitor sample.

### 4.2 Measurement model validation

Following the two-step analytical protocol for Structural Equation Modeling (SEM) advocated by Anderson and Gerbing (1988), the present study conducted Confirmatory Factor Analysis (CFA) to validate the reliability, convergent validity, and discriminant validity of the measurement model before testing the structural relationships among latent constructs.

The overall fit of the measurement model was assessed using multiple widely accepted fit indices, with results indicating an acceptable fit of the CFA model to the observed data: χ^2^ = 1427.024, df = 549, Standardized Root Mean Square Residual (SRMR) = 0.044, Tucker–Lewis Index (TLI) = 0.936, Comparative Fit Index (CFI) = 0.944, Root Mean Square Error of Approximation (RMSEA) = 0.064 (see Table 3). All indices satisfied the established criteria for model fit, suggesting that the measurement model was acceptable and suitable for subsequent structural model testing.

**Table 3:** Model fit indices.

Construct	χ2	*df*	SRMR	TLI	CFI	IFI	RMSEA
Fit indices	1427.024	549	0.044	0.936	0.944	0.944	0.064
Fit criteria	-	-	<0.10	>0.9	>0.9	>0.9	<0.10
Fit status	Y	Y	Y	Y	Y	Y	Y

For the measurement model, χ^2^(549) = 1427.024, *p* < 0.001. The fit indices indicate an acceptable model fit: χ^2^/df = 2.599 (< 3), SRMR = 0.044 (< 0.08), TLI = 0.936 (> 0.9), CFI = 0.944 (> 0.9), IFI = 0.944 (> 0.9), RMSEA = 0.064 (< 0.08). AIC = 228.715, BIC = 691.850.

The reliability and convergent validity of the model were evaluated using three core criteria: standardized factor loadings, Composite Reliability (CR), and Average Variance Extracted (AVE). As demonstrated in Table 4, all standardized factor loadings of the observed variables on their corresponding latent constructs exceeded 0.50 and were statistically significant at the *p* < 0.001 level. This indicates that the measurement items were acceptably associated with their corresponding latent constructs. The CR values for all latent variables ranged from 0.909 to 0.959, all exceeding the recommended threshold of 0.70, suggesting satisfactory internal consistency of the measurement scales. In addition, the AVE values ranged from 0.715 to 0.853, all exceeding the recommended threshold of 0.50. Taken together, these results indicate that the measurement model demonstrates acceptable reliability and convergent validity. Although the factor loadings varied across items, all remained above the recommended minimum threshold, supporting the retention of the measurement indicators in the CFA model.

**Table 4:** Average variance extracted and C.R. for CFA.

Factor (latent variable)	Manifest variable	Coef.	Std. error	CR (critical ratio)	Std. estimate	SMC	AVE	C.R.
RI	RI 1	1			0.837	0.7	0.715	0.909
	RI 2	1.006	0.052	19.262	0.822	0.676		
	RI 3	0.994	0.05	19.942	0.842	0.708		
	RI 4	1.071	0.05	21.341	0.881	0.777		
TA	PRE 1	1			0.983	0.965	0.853	0.959
	PRE 2	0.951	0.024	38.867	0.913	0.833		
	PRE 3	0.931	0.026	36.387	0.9	0.809		
	PRE 4	0.914	0.025	35.903	0.897	0.804		
	IMM 1	1			0.899	0.808	0.812	0.945
	IMM 2	0.971	0.038	25.665	0.874	0.765		
	IMM 3	0.994	0.033	29.776	0.929	0.862		
	IMM 4	0.962	0.035	27.609	0.901	0.813		
	INT 1	1			0.894	0.799	0.832	0.952
	INT 2	0.994	0.035	28.797	0.919	0.845		
	INT 3	1.012	0.035	29.157	0.924	0.853		
	INT 4	0.981	0.035	28.282	0.913	0.833		
SU	SI 1	1			0.855	0.731	0.802	0.924
	SI 2	1.011	0.044	22.872	0.886	0.785		
	SI 3	1.078	0.041	26.101	0.943	0.89		
	HM 1	1			0.997	0.995	0.851	0.945
	HM 2	0.929	0.028	33.321	0.879	0.773		
	HM 3	0.922	0.027	34.579	0.887	0.787		
	HAB 1	1			0.64	0.41	0.736	0.916
	HAB 2	1.464	0.095	15.387	0.977	0.955		
	HAB 3	1.356	0.094	14.495	0.876	0.767		
	HAB 4	1.448	0.098	14.846	0.902	0.814		
VS	FS 1	1			0.862	0.742	0.791	0.938
	FS 2	1.011	0.043	23.45	0.876	0.768		
	FS 3	1.059	0.041	26.153	0.926	0.858		
	FS 4	1.01	0.042	24.266	0.892	0.795		
	ES 1	1			0.996	0.992	0.827	0.935
	ES 2	0.918	0.03	30.712	0.864	0.746		
	ES 3	0.948	0.031	30.54	0.862	0.743		
	PS1	1			0.993	0.987	0.811	0.927
	PS2	0.928	0.033	27.977	0.843	0.71		
	PS3	0.95	0.032	29.438	0.857	0.735		

As demonstrated in Table 5, the Fornell–Larcker criterion was satisfied for all constructs. Although several inter-construct correlations increased following the implementation of the corrected estimation, the square root of AVE for each construct remained greater than its correlations with the other constructs, indicating acceptable discriminant validity.

**Table 5:** Discriminant validity.

Constructs	RI	PRE	IMM	INT	SI	HM	HAB	FS	ES	PS
RI	0.846									
PRE	0.5	0.924								
IMM	0.567	0.69	0.901							
INT	0.564	0.741	0.836	0.912						
SI	0.385	0.395	0.426	0.453	0.895					
HM	0.365	0.377	0.397	0.434	0.721	0.922				
HAB	0.377	0.377	0.417	0.413	0.722	0.718	0.858			
FS	0.530	0.459	0.474	0.458	0.390	0.417	0.413	0.889		
ES	0.458	0.376	0.342	0.406	0.380	0.377	0.384	0.734	0.909	
PS	0.463	0.377	0.405	0.395	0.484	0.448	0.471	0.726	0.691	0.9

It is noteworthy that the correlations between SI, HM, HAB, and FS ranged from 0.390 to 0.417, whereas those between PS and the constructs ranged from 0.448 to 0.484. In more detail, hedonic motivation demonstrated moderate correlations with presence (*r* = 0.377), immersion (*r* = 0.397), and interactivity (*r* = 0.434). These correlations remained well below the square root of AVE for hedonic motivation (0.922) and for the three affordance dimensions, as demonstrated in Table 5. The findings indicate that, while the constructs demonstrate practical relevance, they remain empirically distinguishable within the current model.

### 4.3 Structural model assessment and hypothesis testing

#### 4.3.1 Structural model fit

Before hypothesis testing, the overall fit of the structural model was assessed. The results indicated that the structural model showed an acceptable overall fit to the sample data, with absolute fit indices of χ^2^/df = 2.629, GFI = 0.843, AGFI = 0.820, and RMSEA = 0.065; incremental fit indices of IFI = 0.940, NFI = 0.907, and CFI = 0.940; and parsimonious fit indices of PGFI = 0.735 and PNFI = 0.836. Taken together, these indices suggest that the structural model fit the data at an acceptable level and was suitable for subsequent hypothesis testing through structural path analysis.

#### 4.3.2 Main hypothesis verification

Maximum likelihood estimation in AMOS 26.0 was employed to assess the direct effects between latent constructs (including second-order constructs and their first-order dimensions) in the structural model. The significance of path coefficients was evaluated with Critical Ratio (CR) and *p*-values. A path coefficient was considered statistically significant at the 0.05 level when the absolute value of CR was greater than 1.96. The results of all primary and secondary hypotheses are summarized in Table 6.

**Table 6:** Summary of main hypothesis verification results.

Hypothesis	Path description	*β*	CR	*p*-value	Supported?
H1	TA → VS	0.369	5.958	***	Y
H2	SU → VS	0.391	6.233	***	Y
H3	TA → SU	0.549	8.894	***	Y
H4	VS → RI	0.608	10.675	***	Y

***p < 0.001.

As demonstrated in Table 6, the results of the SEM path analysis supported all four primary hypotheses. Specifically, TA exerted a significant positive effect on VS (*β* = 0.369, CR = 5.958, *p* < 0.001), thereby supporting H1. The SU model exhibited a substantial positive predictive effect on VS (*β* = 0.391, CR = 6.233, *p* < 0.001), thereby supporting H2. TA demonstrated a substantial positive influence on SU (*β* = 0.549, CR = 8.894, *p* < 0.001), supporting H3. Finally, the study found that VS exhibited a robust and significant positive effect on RI (*β* = 0.608, CR = 10.675, *p* < 0.001), supporting H4.

#### 4.3.3 Sub-hypothesis verification

To verify the sub-hypotheses regarding the differential effects of each first-order dimension on the corresponding outcome variables, this study evaluated the validity and relative contribution of each first-order dimension through two core SEM-based criteria. First, we examined the standardized factor loadings of all dimensions on their corresponding second-order latent construct to determine the degree to which the dimension explains the core construct. Second, we evaluated the standardized indirect effects of each dimension on the outcome variable by assessing each dimension’s factor loading on the second-order construct and the structural path coefficients between the second-order construct and the outcome variable. The factor loadings of the first-order dimensions on their respective second-order latent constructs were statistically significant at p < 0.001, as validated in the CFA stage. The results for all sub-hypotheses are summarized in Table 7.

**Table 7:** The results for sub-hypotheses.

Hypothesis	Path description	λ	Standardized indirect effect	*p*-value	Supported?
Sub-hypotheses for H1					
H1-1	PRE → VS	0.790	0.292	***	Y
H1-2	IMM → VS	0.887	0.327	***	Y
H1-3	INT → VS	0.938	0.346	***	Y
Sub-hypotheses for H2					
H2-1	SI → VS	0.858	0.335	***	Y
H2-2	HM → VS	0.838	0.328	***	Y
H2-3	HAB → VS	0.832	0.325	***	Y
Sub-hypotheses for H3					
H3-1	PRE → SU	0.790	0.434	***	Y
H3-2	IMM → SU	0.887	0.487	***	Y
H3-3	INT → SU	0.938	0.515	***	Y
Sub-hypotheses for H4					
H4-1	FS → RI	0.880	0.535	***	Y
H4-2	ES → RI	0.817	0.497	***	Y
H4-3	PS → RI	0.828	0.503	***	Y

***p < 0.001.

#### 4.3.4 Mediation effect analysis

The bias-corrected Bootstrap method with 5,000 repeated resamples was employed to test the hypothesized mediating effects. The presence of a mediation effect was deemed statistically significant if the 95% bias-corrected confidence interval (CI) did not include the value of 0 (Table 8).

**Table 8:** Results of mediation effect analysis.

Mediation path	Total effect	Indirect effect	Direct effect	95% bias-corrected CI (indirect)	Mediating role
TA → SU → VS	0.584	0.215	0.369	[0.140, 0.308]	Partial
SU → VS → RI	-	0.238	-	[0.156, 0.330]	Full

The Bootstrap test results indicated that the indirect effect of TA on VS through SU was 0.215 with a 95% confidence interval of [0.140, 0.308], which did not contain 0. Since the direct effect of TA on VS remained significant, SU was confirmed to play a significant partial mediating role in this relationship.

**Figure 3:**
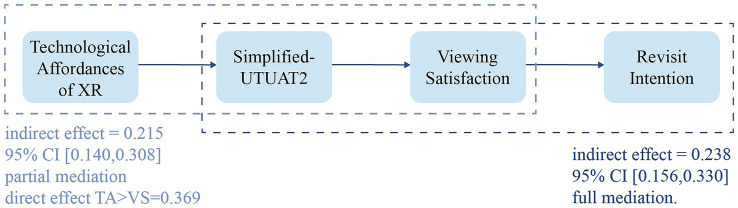
Visual summary of the key mediation effects. The figure summarizes the two mediation paths supported by the bootstrap analysis. The path from technological affordances to viewing satisfaction through simplified UTAUT2 is a significant partial mediation effect. The path from simplified UTAUT2 to revisit intention through viewing satisfaction is a significant full mediation effect.

Subsequent analysis indicated that the indirect effect of SU on RI through VS was 0.238 with a 95% confidence interval of [0.156, 0.330]. This finding indicates that VS significantly mediated the relationship between SU and RI (Figure 3).

## 5 Discussion

This study examines how technological affordances, socio-psychological acceptance, post-experience satisfaction, and revisit intention are linked in XR art exhibitions. By bringing these elements together, it offers a more structured way of understanding how technical design is translated into cultural participation. The results suggest that continuous engagement in XR art exhibitions is shaped not only by sensory immersion alone but also by how technological capabilities are socially interpreted, psychologically accepted, and evaluated after the experience.

### 5.1 Findings

The findings of this study help clarify how XR technological affordances, user acceptance, viewing satisfaction, and revisit intention are connected in the context of art exhibitions. Rather than explaining XR art experience primarily through hardware fidelity and sensory immersion, the present study offers a more structured socio-technical account that links technological affordances, socio-psychological acceptance, post-experience evaluation, and revisit intention.

First, the present findings indicate that both Technological Affordances and the Simplified UTAUT2 framework positively influence viewing satisfaction, with the latter exerting a slightly stronger effect. This finding indicates that viewing satisfaction in XR art exhibitions is influenced not only by the technical capabilities of the system but also by the psychological interpretation and acceptance of the experience by visitors. In this respect, the findings are broadly consistent with previous research showing that technological features usually affect user response through perceptual and evaluative processes rather than through hardware properties alone (Makransky and Lilleholt, 2018; Jeon, 2023). Concurrently, the present findings diverge to a certain extent from those studies that place primary emphasis on immersive atmosphere or artistic-technological innovation in XR art settings (e.g., Lai et al., 2023). In the present study, social influence was identified as the strongest predictor within the Simplified UTAUT2 framework. One potential explanation for this phenomenon is that XR art exhibitions involve a relatively open-ended and interpretive experience. Consequently, visitors may rely more on the opinions and reactions of others when judging the experience’s meaningfulness and worthiness.

Second, all three dimensions of Technological Affordances contributed significantly to both technology acceptance and viewing satisfaction, but their relative effects were clearly different. Interactivity emerged as the strongest dimension, followed by immersion and presence. This finding does not contradict earlier XR research that has emphasized the significance of immersion and presence (e.g., Makransky and Lilleholt, 2018; Cheng et al., 2024). Instead, it introduces a crucial caveat. In the context of exhibitions of XR art, visitors do not appear to respond most strongly to the virtual environment simply because it is immersive. In contrast, it has been observed that visitors appear to respond more strongly when the system enables meaningful action, controllable interaction, and a sense of active engagement with the exhibition. In this sense, the present findings align more closely with research emphasizing the importance of agency and interaction quality in XR engagement (Jeon, 2023). The findings of this study indicate that, within the context of art exhibitions, active engagement may hold more significance than passive sensory absorption.

Third, the hypothesis that viewing satisfaction is the most direct antecedent of revisit intention was confirmed. In the present study, the three dimensions of satisfaction were examined, with the results indicating that functional satisfaction demonstrated the strongest effect, followed by physical satisfaction and emotional satisfaction. This finding is consistent with continuance research, which treats satisfaction as an important post-use predictor of continued intention (Bhattacherjee, 2001). This finding aligns with recent studies on immersive exhibition experiences, which have demonstrated that satisfaction plays a pivotal role in shaping continuance intention following VR art encounters (Cheng et al., 2024). Concurrently, the extant results suggest that satisfaction with XR art exhibitions is not uniform internally. While the emotional response is evidently significant, it appears that the intention to revisit is contingent on the experience being perceived as smooth, manageable, and aligned with the visitors’ viewing requirements. This may help explain why novelty and emotional impact can attract initial interest, but do not necessarily sustain repeat visits, unless the exhibition is also functionally coherent and physically comfortable.

Finally, the mediation results help clarify how these relationships function in conjunction. The partial mediation from Technological Affordances to viewing satisfaction through the Simplified UTAUT2 framework suggests that the effect of XR design on satisfaction is not purely direct. Conversely, it appears that technological affordances become more effective when they are first filtered through visitors’ acceptance-related judgments. The full mediation from the Simplified UTAUT2 framework to revisit intention through viewing satisfaction similarly suggests that socio-psychological acceptance alone does not directly translate into return intention unless it is consolidated into a positive post-experience evaluation. This reading is also consistent with the broader pattern of first-order results in the model, in which interactivity showed the strongest contribution within Technological Affordances, while functional satisfaction showed the strongest contribution within viewing satisfaction. Given that the mediation analysis in this study was conducted at the level of second-order constructs, these more specific links should be understood as theoretical interpretations of the overall pattern, rather than as additional dimension-level mediation tests.

### 5.2 Implications

#### 5.2.1 Theoretical implications

First, this study challenges the long-standing technocentric bias that has historically dominated research in the field of XR user experience. By doing so, it extends the discussion of SDT in non-utilitarian esthetic contexts. Contrary to previous studies, which prioritized hardware fidelity and passive sensory immersion (e.g., Albrezzi, 2024; Lai et al., 2023), our SEM results demonstrated that interactivity, defined as agentic participation, exerts a stronger predictive effect on satisfaction and technology acceptance than presence and immersion. The findings suggest that user autonomy needs, rather than passive visual absorption alone, play a central role in XR esthetic experience. Consequently, it enriches the contextual adaptation of SDT in digital art research.

Second, this study offers a crucial clarification: technological affordances and hedonic motivation should not be treated as interchangeable concepts. Within the domain of XR art, users may encounter profound levels of immersion or perceive significant interactivity without necessarily evaluating this experience as pleasurable. This demonstrates that pleasurable experiences depend on factors beyond mere technological immersion. It is imperative to maintain the separation of these constructs to ensure the preservation of the distinction between system attributes and user evaluations.

Third, this study challenged the “isolationist paradigm” of HMD-based XR and revised the technology acceptance theory in immersive virtual environments. By identifying social influence as the strongest predictor of satisfaction within the simplified UTAUT2 framework, the findings suggest that even in physically segregated virtual environments, art appreciation remains a socially constructed communicative act (Cialdini and Goldstein, 2004). This finding complements the social presence theory in virtual environments, revealing that shared intentionality is still a fundamental human motivation in solitary immersive experiences. Furthermore, it helps clarify the tension between physical isolation and sociality in XR research.

Fourth, this study elucidated the conceptual boundaries between core constructs in XR art research and offers a structured explanatory perspective. A second-order structural equation model analysis was conducted to empirically distinguish the psychological acceptance layer (simplified UTAUT2) and the post-experience evaluation layer (multi-dimensional satisfaction). This approach addressed the conceptual overlap and model inflation issues criticized by prior research. The validated dual mediation model distinguished the stepwise transmission mechanism from technological affordances to sustained behavioral intention, providing a standardized analytical framework for subsequent immersive cultural experience research.

#### 5.2.2 Practical implications

The practical implications of this study should be considered in light of the relative strength of the empirical findings. If the goal is to improve immediate visitor satisfaction, exhibition organizers are advised to prioritize the consideration of social influence and interactivity. If the goal is to strengthen revisit intention, the primary concern should be the functional fluency and physical comfort of the XR experience. These recommendations are most directly applicable to audiences similar to the present sample, namely, relatively young, digitally familiar users who already have some experience with XR exhibition formats.

First, curators and exhibition organizers should recognize that XR art exhibitions should not be regarded as purely individual, headset-based experiences. Given that social influence emerged as the strongest factor within the simplified UTAUT2 framework, institutions should design mechanisms that facilitate evaluation, sharing, and co-experience. In practice, this may include guided entry sessions, small-group onboarding, shared virtual exhibition modes, post-visit discussion prompts, and simple options for visitors to recommend or share exhibition content with others. These interventions are more realistic for museums and cultural institutions than treating “social influence” as an abstract psychological variable. They also directly reflect the finding that visitor satisfaction is partly shaped by how the experience is socially recognized and discussed.

Second, XR exhibition design should prioritize meaningful interactivity over visual immersion alone. Interactivity has been demonstrated to be the strongest contributor among the technological affordance dimensions. Therefore, designers should prioritize the development of responsive forms of participation, rather than passive 360-degree displays. The implementation of such systems need not be complicated; they do not necessitate the use of sophisticated, game-like systems. A more pragmatic approach entails enabling visitors to engage with artworks or exhibition components, select viewing pathways, initiate contextual information, juxtapose divergent visual states, or obtain immediate feedback regarding their actions. The fundamental principle is that interactivity should facilitate interpretation rather than impede it. In the context of art exhibitions, visitors are more likely to report a positive experience when XR allows them to engage in actions that feel meaningful and comprehensible.

Third, for institutions aspiring to foster repeat visits rather than ephemeral, novelty-driven consumption, the principles of system fluency and physical comfort ought to be accorded operational priorities. The strongest predictor of revisit intention was functional satisfaction, followed by physical satisfaction. This finding indicates that revisit intention is contingent not solely on the appeal of the content but also on its ease of navigation, manageability, and physical comfort. Consequently, developers and operators should prioritize the reduction of latency, the establishment of stable spatial mapping, the incorporation of intuitive interaction logic, the provision of clear instructional cues, and the facilitation of rapid recovery from tracking errors. Concurrently, meticulous management of headset fit, weight, hygiene, ventilation, and session duration is imperative to mitigate fatigue and cybersickness. In pragmatic terms, an exhibition that is technically stable and physically comfortable is more likely to generate repeat attendance than one that is impressive but difficult to use.

### 5.3 Limitations and future research

While the present study provides valuable insights, it is not without limitations. First, the study is based on cross-sectional survey data. Even though the proposed relationships are grounded in prior theory, the present design does not allow for strong causal claims. Furthermore, the findings are predominantly reliant on the self-reported evaluations of participants following their experiences of attending an XR art exhibition. While such data may offer valuable insights into overall judgments, they may not fully capture the dynamic nature of moment-to-moment changes in attention, emotional arousal, or physical discomfort during the experience. Future research could address this issue by using longitudinal designs, experimental manipulations, or multimethod approaches that combine survey data with behavioral observation and physiological measures, such as eye-tracking, heart rate variability, or cybersickness-related indicators.

Second, this study adopted a simplified UTAUT2. This choice was made for the specific context of XR art exhibitions, where participation is primarily experience-oriented and where equipment and technical support are typically provided by the hosting institution. However, this simplification also narrows the scope of the model. Performance expectancy, effort expectancy, facilitating conditions, and price value may still matter in other XR settings, especially for first-time users or in contexts where device access, technical support, and cost considerations vary more substantially across users. Consequently, future research may involve a comparison of simplified and full UTAUT2 models across various XR usage settings.

Third, the respondents in this study were primarily from China, concentrated among young and middle-aged groups, and were specifically selected for their prior experience with XR-related art exhibitions. This suggests that the model presented here was tested within an audience that is relatively more digitally proficient and possesses relevant prior experience, rather than among the broader museum-going public. Consequently, the variable relationships observed in this study may appear stronger than they would in a general population. It is recommended that future research seek to provide further validation of this model using samples that are more heterogeneous, including older audiences, first-time visitors to XR exhibitions, and respondents from a range of cultural backgrounds. This would allow for the examination of whether these pathway relationships remain stable across different groups.

In conclusion, the present study examined XR art exhibitions as a broad category, without distinguishing between specific technological modalities or exhibition types. In practice, user experiences may differ considerably across VR, AR, and MR systems, as well as across different exhibition formats such as contemporary art exhibitions, cultural heritage displays, and immersive installations. It is recommended that future research efforts direct their attention toward a more direct comparison of these scenarios. This comparison should determine which components of the model demonstrate robustness across different contexts, and which components are more context-dependent.

## Data Availability

The raw data supporting the conclusions of this article will be made available by the authors, without undue reservation.
