# Supplementary material for: Beyond immersion: disentangling the technological and social drivers of visitor satisfaction in XR art exhibitions
Source: Front Psychol. 2026 Apr 1;17:1793811. doi: 10.3389/fpsyg.2026.1793811 (PMC13079572; doi:10.3389/fpsyg.2026.1793811)
Supplement: Supplementary file 1 [file Data_Sheet_1.PDF]

TABLE S1 Measurement items (Chinese version)

Constructs		Items	Contents
**Technological Affordances（TA）**	**Presence (PRE)**	PRE 1	我能够清晰感知到 XR 艺术展览的三维空间特性，而非仅作为平面屏幕上的二维影像。
		PRE 2	观展过程中带来的真实感体验让我觉得虚拟人物/物体是真实存在的。
		PRE 3	XR 艺术展中不存在与环境不符的事物让我混淆。
		PRE 4	我能清晰地感知到 XR 艺术展中所发生的事情。
	**Immersion (IMM)**	IMM 1	我更专注于艺术展本身而不是任何外部干扰。
		IMM 2	我对展览的进展情况感到好奇。
		IMM 3	我发现自己在使用 XR 技术时很容易忘记自己是在观看展览。
		IMM 4	观览活动期间，时间过得很快。
	**Interactivity (INT)**	INT 1	我感觉我能接触到展览中的虚拟物体。
		INT 2	我能通过五感（视觉、听觉、嗅觉等）感知艺术展的内部环境。
		INT 3	我经常感到兴奋，因为我觉得自己是展览的一部分。
		INT 4	我非常投入观展，有时我想直接与虚拟人物/物体互动。
**Simplified UTAUT2 (SU)**	**Social Influence (SI)**	SI 1	对我而言重要的人认为我应该使用 XR 技术来观看艺术展。
		SI 2	影响我行为的人认为我应该使用 XR 技术来观看艺术展。
		SI 3	我重视其意见的人希望我使用 XR 技术来观看艺术展。
	**Hedonic Motivation (HM)**	HM 1	使用 XR 技术观看艺术展很有趣。
		HM 2	使用 XR 技术观看艺术展令人感到放松。
		HM 3	使用 XR 技术的艺术展非常有趣。
	**Habit (HAB)**	HAB 1	使用 XR 观看艺术展已成为我的一种习惯。
		HAB 2	我沉迷于使用 XR 技术观看艺术展。
		HAB 3	我必须使用 XR 观看艺术展。
		HAB 4	使用 XR 观看艺术展对我来说已经成为一种习以为常的事情。

**Viewing Satisfaction (VS)**	**Functional Satisfaction （FS）**	FS 1	我认为在艺术展览中使用的 XR 技术操作简单且流畅。
		FS 2	我认为展览中使用XR技术能够清晰传递艺术作品的核心信息。
		FS 3	我认为展览中 XR 互动功能可以显著提升参观过程中的趣味性。
		FS 4	我认为展览中 XR 技术提供的交互内容与我的观展需求高度匹配。
	**Emotional Satisfaction (ES)**	ES 1	在艺术展中使用 XR 技术让我很开心。
		ES 2	我会向朋友推荐 XR 艺术展览。
		ES 3	XR 技术激励我想要了解并参与更多艺术展览。
	**Physical Satisfaction (PS)**	PS 1	在 XR 艺术展观览过程中，我的身体并未感到明显不适。
		PS 2	在 XR 艺术展观览过程中，并未让我长期保持固定姿势。
		PS 3	在 XR 艺术展观览过程中，我并未感觉到疲劳。
**Revisit Intention (RI)**		RI 1	我愿意将来再去 XR 艺术展。
		RI 2	我总是把使用 XR 技术的艺术展作为首选。
		RI 3	如果可以，我还会再去这种 XR 艺术展。
		RI 4	我会和更多的同伴一起去 XR 艺术展。
